# Immune Checkpoint Inhibitors in Melanoma and HIV Infection

**DOI:** 10.2174/1874613601711010091

**Published:** 2017-11-14

**Authors:** Antonio Marra, Giosuè Scognamiglio, Ilaria Peluso, Gerardo Botti, Celeste Fusciello, Amelia Filippelli, Paolo A. Ascierto, Stefano Pepe, Francesco Sabbatino

**Affiliations:** 1 Department of Medical Oncology, San Gerardo Hospital, via G. B. Pergolesi, 20052 Monza, Italy; 2 Pathology Unit, Istituto Nazionale per lo Studio e la Cura dei Tumori “Fondazione G. Pascale”, via M. Semmola, 80131 Naples, Italy; 3 Hematology Unit, Department of Clinical and Surgical Medicine, University of Naples Federico II, via S. Pansini, 80131 Naples, Italy; 4 Oncology Unit, Department of Medicine, Surgery and Dentistry, University of Salerno, via Allende, 84081 Baronissi (Salerno), Italy; 5 Pharmacology Unit, Department of Medicine, Surgery and Dentistry, University of Salerno, via Allende, 84081 Baronissi (Salerno), Italy; 6 Unit of Melanoma, Cancer Immunotherapy and Innovative Therapy, Istituto Nazionale per lo Studio e la Cura dei Tumori “Fondazione G. Pascale”, via M. Semmola, 80131 Naples, Italy

**Keywords:** Melanoma, HIV, Immunotherapy, Immune checkpoint molecules, Infection, Survival

## Abstract

**Introduction:**

Immunotherapy with immune checkpoint inhibitors increases the overall survival of patients with metastatic melanoma regardless of their oncogene addicted mutations. However, no data is available from clinical trials of effective therapies in subgroups of melanoma patients that carry chronic infective diseases such as HIV. Evidences suggest a key role of the immune checkpoint molecules as a mechanism of immune escape not only from melanoma but also from HIV host immune response.

**Conclusion:**

In this article, firstly, we will describe the role of the immune checkpoint molecules in HIV chronic infection. Secondly, we will summarize the most relevant clinical evidences utilizing immune checkpoint inhibitors for the treatment of melanoma patients. Lastly, we will discuss the potential implications as well as the potential applications of immune checkpoint molecule-based immunotherapy in patients with melanoma and HIV infection.

## 1 BACKGROUND

Melanoma is an aggressive form of skin cancer characterized by poor prognosis and high mortality. In Europe, about 100,000 new cases of melanoma are diagnosed every year and incidence of melanoma is continuously increasing [1]. Patients with immunodeficiency and especially those affected by Human Immunodeficiency Virus (HIV) chronic infection are characterized by a higher risk of tumor development including melanoma. The incidence of melanoma in HIV infected patients is 2.6 higher as compared to no HIV patients [2]. This increased incidence reflects both a decreased efficiency of the host immune response in eliminating potentially malignant cells and an improvement in the treatment of HIV patients because of the development of new antiretroviral agents. The latter prolong the survival of the infected patients [3] increasing the time of immunodeficiency and the possibility of tumor development. However, in HIV patients, melanoma shows a more aggressive phenotype and poorer survival outcomes as compared to non HIV patients [4]. Implementation of monoclonal antibodies (mAbs), inhibiting the interaction of immune suppressive checkpoint molecules with their ligands, has dramatically changed the clinical course of cancer patients, including those with melanoma. The administration of mAbs targeting immune checkpoint molecules such as Cytotoxic T Lymphocyte Antigen-4 (CTLA-4) and Programmed Death-1 (PD-1) significantly increases overall survival (OS) of metastatic melanoma patients [5]. In addition, novel mAbs targeting different immune checkpoint molecules, such as Programmed Death Ligand-1 (PD-L1), T-cell immunoglobulin and mucin-domain containing-3 (TIM-3), Lymphocyte Activation Gene-3 (LAG-3) and T cell immunoreceptor with Ig and ITIM domains (TIGIT), are now being tested in promising clinical trials alone or in combination with CTLA-4 or PD-1 inhibitors. Nevertheless, scant information is available about the efficacy and safety of these therapeutic strategies in HIV infected melanoma patients. Indeed, HIV infected melanoma patients are currently excluded from novel clinical trials because of their immunodeficient status, the potential drug interactions and the effects of HIV infection on the safety and activity of the investigational agents. These findings and the lack of curative therapy for HIV infected melanoma patients with metastatic disease emphasize the urgent need to define novel effective therapies for this subgroup of melanoma patients.

*In vitro* and *in vivo* evidences suggest a major role of immune checkpoint molecules in the pathogenesis and clinical progression of HIV infection. PD-1/PD-L1, CTLA-4, TIM-3, LAG-3 and TIGIT are higher expressed on the lymphocytes of HIV-positive as compared to HIV-negative patients [6-12]. However, the role of immune checkpoint molecules as well as the potential application of immune checkpoint targeting strategies in HIV disease still needs to be better defined.

In this article, firstly, we will describe the role of CTLA-4, PD-1, PD-L1, TIM-3, LAG-3 and TIGIT during HIV infection. Secondly, we will summarize the most relevant clinical evidences utilizing immune checkpoint blockade for the treatment of metastatic melanoma patients. Lastly, we will discuss the potential implications as well as the potential application of immune checkpoint-based immunotherapy in patients with melanoma and HIV.

## 2 ROLE OF IMMUNE CHECKPOINT MOLECULES IN HIV INFECTION

**Fig. (1):**
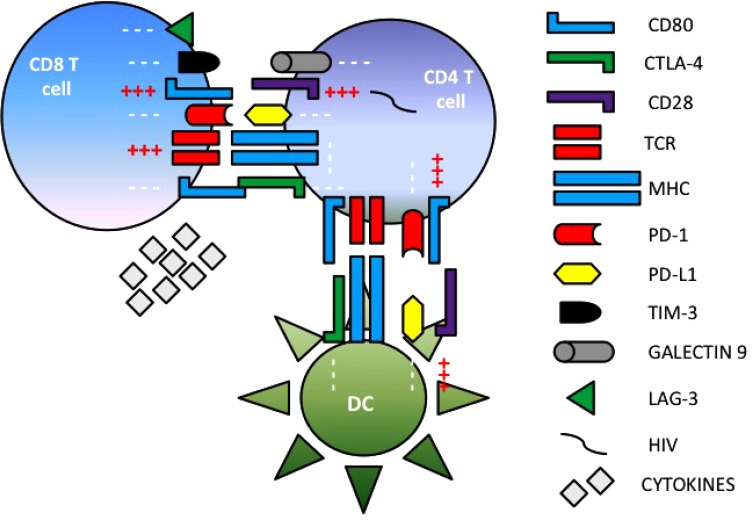
**Immune checkpoint molecule expression in HIV infection.** Immune checkpoint molecules can influence HIV chronic persistence by inhibiting immune system activation and elimination of HIV infected cells.

Many *in vitro* and *in vivo* studies have been performed to define the interactions between HIV disease and immune checkpoint molecules. PD-1, PD-L1, CTLA-4, TIM-3, LAG-3 and TIGIT have been involved in chronic viral persistence and are usually used as a marker to define exhausted T cells during HIV infection (**Fig. 1**) [6-12]. In addition, T-cell exhaustion markers such as PD-1, TIM-3 and LAG-3, measured prior to antiretroviral therapy, are often used to strongly predict time of viremia rebound [9].

CTLA-4 gene polymorphisms and their involvement in chronic viral infection were described for the first time in Hepatitis B Virus (HBV) infection [13]. In HIV subjects, CTLA-4 is significantly higher on CD4+ T cells as compared to cells from normal donors. Furthermore, CTLA-4 levels are negatively correlated with both CD4+ T cell number and CD4/CD8 ratio, while are positively correlated with HIV viral load and disease progression [14, 15]. CTLA-4 is also expressed by HIV-specific CD4+ T cells although its levels change based on the timing of HIV infection [14-16]. Specifically, CTLA-4 upregulation on CD4+ T cells is followed by its downregulation during disease progression. CTLA-4 downregulation is mediated by the Negative Regulatory Factor (Nef), a protein involved in HIV survival and viral replication into T cells [16].

The axis of PD-1 and PD-L1 can also modulate HIV-specific T cell response although contrasting data are reported in the literature about the correlation of PD-1 expression with number of CD4+ T cells, HIV viral load and disease progression. PD-1 is overexpressed on both CD4+ and CD8+ T cells of HIV patients. In those, CD4+ and CD8+ T cells express significantly higher levels of PD-1 as compared to cells from normal donors [17, 18]. In addition, PD-1 levels are negatively correlated with CD4+ T cell number as well as with CD4/CD8 ratio, while are positively correlated with both HIV viral load and disease progression [17-20]. PD-1 levels on CD4+ T cells are also negatively associated with the viral replication *in vivo* [21] although Chomont et al. reported that infected CD4+ T cells co-expressing PD-1 might represents a major reservoir of HIV [22]. Lastly, PD-L1 is significantly elevated on monocytes and B cells in the peripheral blood of HIV-infected individuals as compared to HIV-negative controls. Its expression negatively correlated with the number of CD4+ T cells and its levels are associated with both viral load and disease progression [23].

Several mechanisms can regulate PD-1 and PD-L1 expression in T cells from HIV infected patients. The common gamma-chain cytokines including IL-2, IL-7, IL-15 and IL-21 upregulate both PD-1 and PD-L1 *in vitro* [24]. In addition, the accessory HIV protein Nef upregulates PD-1 through p38 MAPK-dependent mechanism [25].

The immune checkpoints TIM-3, LAG-3 and TIGIT have been also investigated in the pathogenesis of HIV. TIM-3 expression on CD8+ T cells is increased in HIV patients as compared to uninfected subjects. Furthermore, TIM-3 upregulation positively correlates with HIV viral load and CD38 expression, while it is negatively associated with CD4+ T cell number [26]. Co-expression of TIM-3 and PD-1 is associated with a more severe exhaustion of T cells during HIV infection *in vitro* [27]. The ligand of TIM-3, galectin-9, is rapidly released during acute HIV infection and galectin-9-TIM-3 crosstalk contributes to persistent T cell dysfunction [28]. In contrast to this data, Hoffmann et al. showed that TIM-3 expression might be a protective biomarker in some infected subjects because of its association with a delayed HIV disease progression [12].

LAG-3 expression on CD8+ T cells is associated with HIV plasma viral load, but not with number of CD4+ T cell [12]. Upregulation of LAG-3 on both CD4+ and CD8+ T cells is correlated with HIV disease progression and a prolonged antiretroviral therapy can reduce its expression. In addition, the overexpression of LAG-3 on T cells or the stimulation of LAG-3 on T cells leads to a reduction of T cell responses [29].

TIGIT is upregulated on CD8+ T cells during HIV infection and the co-expression of PD-1 and TIGIT positively correlates with HIV disease progression [10]. Tauriainen et al. showed that an increased TIGIT expression i*n vitro* correlates with a decreased functional capacity of HIV-specific CD8+ T cells [30]. Lastly, LAG-3 and TIGIT, alone or in combination with PD-1, positively correlate with an increased number of CD4+ T cells that harbor an integrated HIV DNA [11].

Few experimental evidences have been testing the potential applicability of immune checkpoint inhibitors in HIV infection and contrasting results are reported in the literature. Blocking of both PD-1 and CTLA-4 in HIV-1-specifìc CD4+ and CD8+ T cells leads to a recovery of cell proliferation and a cytokine production *in vitro* [19]. Furthermore, CTLA-4 blockade by an anti-CTLA-4 mAb increases CD4+ T cell proliferation and augments HIV-specific CD4+ T cell function *in vitro* [14, 15]. In a simian immunodeficiency virus (SIV)-infected macaque model, administration of an anti-CTLA-4 mAb decreases viral replication of the infected subjects while it is associated with an increased viral replication at mucosal site and no benefit in terms of plasma viral load and survival [31, 32]. Into the clinical setting just few case reports have been reported. Wightman et al. have recently shown that treatment with anti-CTLA-4 mAb in a metastatic melanoma patients could reactivate HIV from latency [33]. Sabbatino et al. have been reported a melanoma tumor response associated with a decreased viral replication and an increased number of CD4+ T cells in a patient with both HIV infection and metastatic melanoma during treatment with an antiretroviral therapy and an anti-CTLA-4 mAb [34]. Blockade of PD-1 has also been reported in chronic viral infection. *In vitro* blockade of PD-1, in patients affected by HBV, leads to an increased T cell survival as well as to an increased cytokine production, especially in patients with HIV co-infection [35]. Moreover, Trautmann et al. reported that PD-1 blockade enhances the capacity of HIV-specific CD8+ T cells to survive and proliferate leading to an increased production of cytokines and cytotoxic molecules in response to cognate antigen *in vitro* [19]. Even more, *in vitro* stimulation of CD28 in combination with PD-1 blockade synergistically increases HIV-specific CD4+ T cell proliferation [8]. Lastly, in a SIV-infected macaque model, blockade of PD-1 by an anti-PD-1 mAb increases the number of virus-specific CD4+ T cells and memory B cells as well as the levels of envelope-specific antibodies. These immunological effects are associated with the lack of side effects and a significantly increase of OS of the treated SIV-infected macaques [36].

Besides CTLA-4 and PD-1, also blockade of other checkpoint molecules has been tested in chronic viral infection. *In vitro* blockade of TIM-3 signaling pathway enhances the cytotoxic capabilities of HIV specific CD8+ T cells from chronic progression by increasing their functions and their ability to suppress HIV infection of CD4+ T cells [37]. Furthermore, the *ex vivo* blockade of LAG-3 significantly augments HIV-specific CD4+ and CD8+ T cell responses [29]. Lastly, *in vivo* combinatorial blockade of PD-L1 and TIGIT restores viral-specific CD8+ T cell effector response [10].

## 3 IMMUNE CHECKPOINT INHIBITORS IN MELANOMA

The introduction of immune checkpoint inhibitors into clinical setting has drastically changed the survival of metastatic melanoma patients. Several mAbs have been developed to inhibit the interaction of immune regulatory checkpoint molecules CTLA-4 and PD-1 with their ligands CD80 or CD86 and PD-L1 or PD-L2, respectively [38]. As a result, T cells can proliferate and elicit the host immune response against cancer cells.

Ipilimumab (Yervoy, Bristol-Myers Squibb), a fully human immunoglobulin G1 (IgG1), targeting CTLA-4, was the first mAb to demonstrate a survival benefit in patients with metastatic melanoma. In a Phase III randomized clinical trial (MDX010-020) administration of ipilimumab in combination with glycoprotein 100 (gp100) peptide increased OS as compared to gp100 vaccination alone (10.1 versus 6.4 months) [39]. In another Phase III randomized clinical trial (CA184-024), ipilimumab in combination with dacarbazine demonstrated a significantly longer OS as compared to dacarbazine alone (11.2 versus 9.1 months) [40]. An update analysis has confirmed the survival benefit of ipilimumab in metastatic melanoma patients showing an 18.2% of 5-year survival rate for patients treated with ipilimumab plus dacarbazine as compared to 8.8% of patients treated with placebo plus dacarbazine [41]. Even more administration of ipilimumab at 10 mg/kg significantly increased OS of melanoma patients as compared to standard dose of 3 mg/kg (15.7 versus 11.5 months) [42]. However, the relevant results obtained with ipilimumab have been mitigated following the publication of the clinical trials data of the anti-PD-1 mAbs, nivolumab (Opdivo, Bristol-Myers Squibb) and pembrolizumab (Keytruda, Merck), in metastatic melanoma patients.

In a Phase III randomized clinical trial (CheckMate 066) administration of nivolumab, a fully human IgG4 anti-PD-1, improved 1- and 2-year OS rate as compared to standard chemotherapy with dacarbazine in previously untreated patients with metastatic melanoma without BRAF mutation (73.0% versus 41.0% at 1 year and 56.7% versus 26.7% at 2 years) [43, 44]. In another Phase III randomized clinical trial (CheckMate 037) nivolumab demonstrated a higher percentage of overall response rate (ORR) as compared to investigator’s choice chemotherapy in patients with metastatic melanoma who experienced disease progression following anti-CTLA-4 or BRAF inhibitor treatment (31.7% vs 10.6%) [45]. In a Phase II randomized clinical trial (KEYNOTE-002) administration of pembrolizumab, a humanized IgG4 anti-PD-1, at two different doses, was compared to investigator’s choice chemotherapy in metastatic melanoma patients who experienced disease progression after treatment with ipilimumab and/or BRAF inhibitor and/or MEK inhibitor. The 6-month progression free survival (PFS) rate was 34% and 38% for pembrolizumab at 2 and 10 mg/Kg, respectively, while it was only 16% for the chemotherapy group [46]. Median OS was 13.4 and 14.7 months for 2 and 10 mg/kg of pembrolizumab, respectively, while it was 11.0 months for chemotherapy. Eighteen-month OS rates were 40%, 44% and 36% and 24-month rates were 36%, 38% and 30% [47]. Lastly, in a Phase III randomized clinical trial (KEYNOTE-006), pembrolizumab, at two different schedules of treatment (10 mg/Kg every two or three weeks), demonstrated an improvement in PFS (12-month PFS 39% and 38% versus 19%; 24-month PFS 31% and 28% versus 14%) and OS (1-year OS rate 74% and 68% versus 59%; 2-year OS rate 55% and 55% versus 43%) as compared to ipilimumab alone [48, 49].

Immune checkpoint blockade using anti-PD-L1 mAbs is another promising approach for the treatment of melanoma patients with metastatic disease. BMS-956559 (Bristol-Myers Squibb), a fully human IgG4, was the first anti-PD-L1 mAb to show objective tumor responses in patients with solid tumors [50]. In addition, anti-PD-L1 mAbs such as Atezolizumab (MPDL3280A, Roche Genentech), Durvalumab (MEDI4736, Astrazeneca) and Avelumab (MSB00107185, EMD Serono/Merck KGaA/Pfizer) are currently tested in clinical trials and their results are expected soon. Moreover, several ongoing clinical trials are now testing blockade of different checkpoint inhibitors such as LAG-3, TIM-3 or TIGIT, alone or in combination with anti-PD-1/PD-L1 drugs, in metastatic melanoma patients.

Both anti-CTLA-4 and anti-PD-1/PD-L1 mAbs are revolutionizing the clinical approach to melanoma patients regardless the mutational status. However, as we have previously described, the efficacy of this novel immunotherapeutic strategy is limited to up to 40% of treated patients and there is still the need to identify potential predictive biomarkers of treatment response. Several biomarkers are under investigation including PD-L1, PD-L2, FAS, HLA class I and HLA class II antigen expression, immune checkpoints LAG-3, TIM-3, IDO, OX40, CD137 and CD40 expression, tumor infiltrating lymphocytes (TIL), CD4+, CD8+, granzyme B+, CD56+ and FOXP3+ cells, secreted molecules IL-2, IFN-γ, IL-10, IL-4, CXCL9, CXCL10, CCL5, cancer cell mutational load, antigenic peptide expression, gene expression and TCR signaling analysis. So far, none of these biomarkers including PD-L1 expression has been shown to play a major role as a predictive biomarker of response to immune checkpoint molecule based immunotherapy. In a large meta-analysis, which summarized the results of clinical trials utilizing anti-PD-1 mAbs in malignant diseases including melanoma, PD-L1 expression by tumor cells correlated with ORR. However, in both PD-L1 positive and negative tumors clinical response rates and an increased OS were reported [51]. As a result, PD-L1 expression might be used to identify patients that benefit more from anti-PD-1 based immunotherapy but it cannot be used to exclude patients from the treatment with this type of therapy. The combination of different immune checkpoint inhibitors is currently tested as an alternative strategy to increase the ORR and OS of treated melanoma patients.

In a randomized Phase III clinical trial (CheckMate 067), administration of ipilimumab and nivolumab was compared to single agent alone (ipilimumab or nivolumab) in previously untreated melanoma patients. The median PFS was 11.5 months in the nivolumab-plus-ipilimumab group as compared to 6.9 months for nivolumab alone and 2.9 months for ipilimumab alone [52]. The median OS had not been reached in the nivolumab-plus-ipilimumab group and was 37.6 months in the nivolumab group and 19.9 months in the ipilimumab group. The 3-years OS was higher in the nivolumab-plus-ipilimumab group as compared to nivolumab and ipilimumab groups (58% versus 52% and 34%, respectively). However, a higher rate of grade 3-4 immune-related toxicities was reported in the combination group as compared to single agents alone (59% in the nivolumab-plus-ipilimumab group, 21% in the nivolumab group and 28% in the ipilimumab group) [53].

Lastly, immune checkpoint inhibitors have been tested not only in the metastatic setting of melanoma patients, but also as an adjuvant strategy following surgery for high risk melanoma patients. In a Phase III randomized clinical trial the anti-CTLA-4 mAb ipilimumab increased the 5-years rates of recurrence-free survival (40.8% versus 30.3%), OS (65.4% versus 54.4%) and distant metastasis–free survival (48.3% versus 38.9%) as compared to placebo in high-risk stage III melanoma patients [54]. However, also in this case, side effects were not irrelevant. Recently, another Phase III randomized clinical trial compared 1-year administration of nivolumab with ipilimumab in completely resected stage III-IV melanoma patients. Nivolumab significantly improved 12-month rate of recurrence-free survival as compared to ipilimumab (70.5% versus 60.8%), with a reduced incidence of treatment-related grade 3-4 adverse events (14.4% versus 45.9%) [55].

## CONCLUSION

The implementation of immune checkpoint-based immunotherapy is completely revolutionizing the clinical approach to cancer patients. In melanoma, a tumor that for many years has shown high rate of deaths because of its resistance to standard therapy, administration of both anti-CTLA-4 and anti-PD-1 mAbs significantly increases response rates, PFS and OS of the treated patients [38-53].

Nevertheless, in cancer patients carrying a chronic viral infection such as HIV, the anti-tumor activity of these molecules has not been extensively evaluated and clinical trials are still warranted. Some clinical cases showed clinical and immunological response to checkpoint inhibitors in melanoma patients with HIV, HBV or Hepatitis C Virus (HCV) infections [33, 34, 56-60]. Moreover, in a retrospective analysis of 44 patients affected by metastatic tumors (including 29 melanoma patients) and concurrent solid organ transplant, HIV, HBV or HCV infections, the administration of anti-PD-1/PD-L1 mAbs appeared to have clinical activity in the absence of adverse effect on the viral control [61]. Recently, another retrospective study evaluated the efficacy of immune checkpoint blockade in metastatic melanoma patients with concomitant HIV infection, pointing out similar results [62]. Globally, these data provide evidences about the efficiency of immune checkpoint inhibitors as treatment of melanoma and HIV disease. As previously described, HIV infection plays a crucial role in determining the worst prognosis of HIV-infected cancer patients because of the induction of a chronic and progressive immunodeficient status [4]. The latter causes the inability to mount an effective host immune response and the persistence and/or the progressive expression of different immune checkpoint molecules (CTLA-4, PD-1/PD-L1, TIM-3, LAG-3, TIGIT) lastly leads to an immune exhausted phenotype [63-65].

Ideally, treatment of metastatic cancer in patients with HIV should not further compromise immune competence, interact adversely with antiretroviral agents or increase the risk of tumor development. This hypothesis is supported by the results obtained from two recently published Phase I/II clinical trials. In a first trial, patients with advanced hepatocellular carcinoma were treated with nivolumab including those affected by HCV and HBV infection. The results demonstrate that infected patients have similar outcomes in terms of tumor response and safety profile as compared to non-infected subjects [66]. In a second trial, patients with squamous cell carcinoma of the anal canal were treated with nivolumab. A sub-analysis confirmed nivolumab efficacy and safe in both HIV-negative and HIV-positive patients [67, 68]. However, these preliminary data are referred to small cohorts of cancer patients with chronic viral infections and they have to be interpreted cautiously.

Several Phase I/II clinical trials (NCT02408861, NCT03304093, NCT02595866) testing the administration of checkpoint inhibitors, alone or in combination, in patients with HIV and advanced solid tumors are currently ongoing. Their results will shed some light on the efficacy and safety of this therapeutic strategy in this subgroup of cancer patients which has always been excluded from previous clinical trials. In conclusion, there is an urgent need to design new clinical trials in order to determine the effectiveness of treatment with checkpoint molecule inhibitors for the treatment of HIV and/or HIV-related cancer patients.

## LIST OF ABBREVIATIONS

CTLA-4: = Cytotoxic T Lymphocyte Antigen-4;
gp100: = Glycoprotein 100;
HBV: = Hepatitis B Virus;
HCV: = Hepatitis C Virus;
HIV: = Human Immunodeficiency Virus;
HLA: = Human Leukocyte Antigen;
IgG1: = Human Immunoglobulin G1
LAG-3: = Lymphocyte-Activation Gene-3;
mAb: = Monoclonal Antibody;
Nef: = Negative Regulatory Factor;
ORR: = Overall Response Rate
OS: = Overall Survival;
PD-1: = Programmed Death-1;
PD-L1: = Programmed Death-Ligand-1;
PFS: = Progression Free Survival;
SIV: = Simian Immunodeficiency Virus;
TIGIT: = T cell Immunoreceptor with Ig and ITIM Domains;
TIM-3: = T-cell Immunoglobulin and Mucin-domain Containing-3.
